# SARS-CoV-2 B.1.619 and B.1.620 Lineages, South Korea, 2021

**DOI:** 10.3201/eid2802.211653

**Published:** 2022-02

**Authors:** Ae Kyung Park, Il-Hwan Kim, Heui Man Kim, Hyeokjin Lee, Nam-Joo Lee, Jeong-Ah Kim, SangHee Woo, Chae young Lee, Jaehee Lee, Sae Jin Oh, JeeEun Rhee, Cheon-Kwon Yoo, Eun-Jin Kim

**Affiliations:** Korea Disease Control and Prevention Agency, Cheongju, South Korea

**Keywords:** COVID-19, SARS-CoV-2, severe acute respiratory syndrome coronavirus 2, viruses, respiratory infections, zoonoses, B.1.619, B.1.620, South Korea, coronavirus disease, *Suggested citation for this article*: Park AK, Kim I-H, Kim HM, Lee H, Lee N-J, Kim J-A, et al. SARS-CoV-2 B.1.619 and B.1.620 lineages, South Korea, 2021. Emerg Infect Dis. 2022 Feb [*date cited*]. https://doi.org/10.3201/eid2802.211653

## Abstract

We report the rapid emergence of severe acute respiratory syndrome coronavirus 2 lineages B.1.619 and B.1.620 in South Korea. The surge in frequency in a relatively short time emphasizes the need for ongoing monitoring for new lineages to track potential increases in transmissibility and disease severity and reductions in vaccine efficacy.

Since the emergence of coronavirus disease (COVID-19) in December 2019, ≈2 million genomes of SARS-CoV-2 have been sequenced worldwide, revealing that the virus is continuously mutating (*1*). The mutations of SARS-CoV-2 spike protein must be monitored because of its vital role in attaching to the host cell-surface receptor, angiotensin-converting enzyme 2 (ACE2) (*2*), which increases its infectivity (*3*).

As of May 31, 2021, the World Health Organization (WHO) reported the appearance of several variants of concern (VOCs) whose characteristics have serious implications on public health: Alpha (B.1.1.7), Beta (B.1.351), Gamma (P.1), and Delta (B.1.617.2) (*4*). These variants arose from changes in the spike protein, especially in the receptor-binding domain (RBD). The RBD plays an important role in direct interaction with human ACE2; the 4 variants contain >1 of the specific substitutions (K417N, L452R, T478K, E484K, and N501Y) that affect viral fitness and transmissibility. These variants possess substantially higher transmissibility, evade immunity, increase disease severity, reduce vaccine efficacy, and escape diagnostic detection (*5*). WHO designated Lambda (C.37) as a variant of interest (VOI) on June 14, 2021, and Mu (B.1.621) as a VOI on August 30, 2021. These variants were considered likely to become highly transmissible and evade vaccine protection, thus threatening South America, where they were first identified (Acevedo et al., unpub. data, https://doi.org/10.1101/2021.06.28.21259673) (*6*).

Genomic surveillance and open data sharing of viral genome sequences have enhanced near–real-time detection, comparison, and tracking of SARS-CoV-2 variants (*1*). The Korea Disease Control and Prevention Agency (KDCA) has been conducting whole-genome sequencing (WGS) of SARS-CoV-2 in South Korea since the beginning of the pandemic; targeted sequencing of the spike protein is being implemented to strengthen new variant monitoring. Lineage distribution analysis in South Korea indicated that, from its discovery in March 2020 until January 2021, the B.1.497 lineage was predominant in domestic cases (*7*,*8*). B.1.497 formed 1 of 4 major clusters in South Korea but was the only one that had expanded and predominantly circulated in the country (*9*). Sequence analysis indicated that it was originally clustered with North American viruses; additional genetic mutations in this cluster are H1113Y, T2408N, P4223S, and A5770S in open reading frame 1ab (ORF1ab) and Q52H, A222V, E556K, T716I, and A1070V in the spike protein. However, changes have been observed in lineage distribution since March 2021; increases in B.1.619 and B.1.620 are of note. We report the sudden emergence of these 2 lineages harboring the E484K mutation in the spike protein, which rapidly outcompeted the existing variants in South Korea.

## The Study

We collected nasopharyngeal and oropharyngeal swab samples from patients with SARS-CoV-2 cases confirmed by real-time reverse transcription PCR (rRT-PCR). We prepared WGS libraries using QIAseq SARS-CoV-2 Primer Panel and QIAseq FX DNA Library UDI Kit (QIAGEN, https://www.qiagen.com) and sequenced them on MiSeq (Illumina, https://www.illumina.com) with 2 × 150 bp using MiSeq reagent kit version 2 (Illumina). For phylogenetic tree analysis, we aligned whole genomic sequences using MAFFT version 7 (https://mafft.cbrc.jp/alignment/server), inferred maximum-likelihood phylogenetic trees with FastTree version 2.1.9 (http://www.microbesonline.org/fasttree), and visualized using Interactive Tree of Life version 5 (https://itol.embl.de). The sequences have been uploaded to the GISAID EpiCoV database (https://www.gisaid.org).

**Figure 1:**
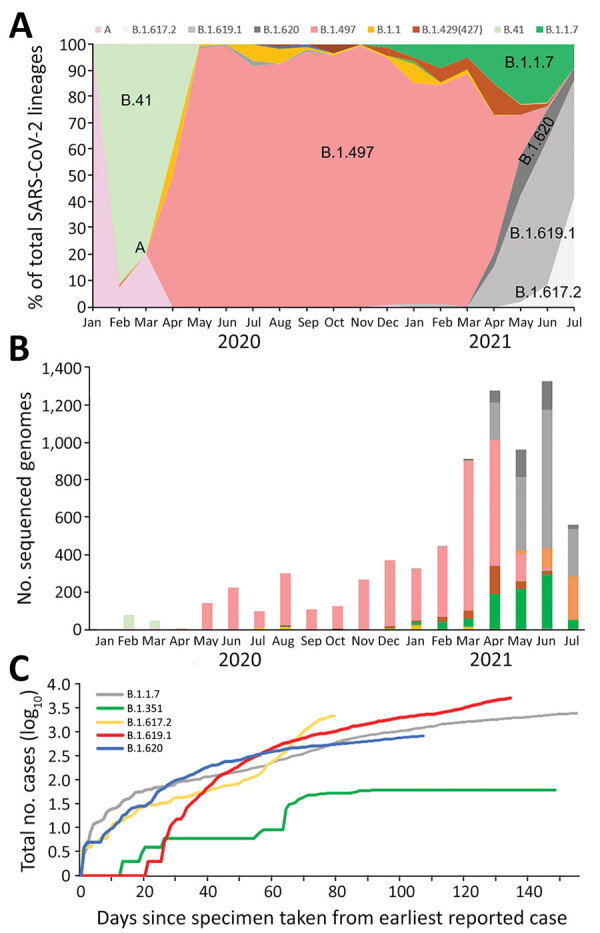
Investigation of SARS-CoV-2 B.1.619 and B.1.620 lineages, South Korea, 2021. A) Distribution of 7 SARS-CoV-2 lineages in domestic cases over time. Data are shown for lineages A, B.41, B.1.497, B.1.1.7 (Alpha variant), B.1.617.2 (Delta variant), B.1.619.1, and B.1.620. B) Number of sequenced genomes over time, by lineage. C) Logarithmic graph of cumulative cases of variants indexed by days since the first reported case as of July 21, 2021. SARS-CoV-2, severe acute respiratory syndrome coronavirus 2.

We obtained WGS of 9,554 SARS-CoV-2 as of July 21, 2021, representing >5% of the total reported positive cases during this period. Specifically, we obtained WGS for 6.2% of the total positive cases during April–July, when the number of infections by B.1.619 and B.1.620 increased sharply. Of those, 7,585 were domestic cases. As described previously (*7*), the A and B.41 lineages were the most prevalent at the beginning of the pandemic in South Korea. However, B.1.497 (formerly known as B.1.3.1) gained predominance in South Korea after its emergence in March 2020 (*8*,*9*). The lineage distribution has been changing since January 2021 as VOCs have emerged. The earliest recorded Alpha variant in South Korea was identified on December 22, 2020; its prevalence increased to 22.0% as of June 2021 (Figure 1). Simultaneously, prevalence rates of B.1.619 and B.1.620 increased rapidly; prevalence was 55.4% in March 2021 and 11.5% in June 2021. The increase in the prevalence of these 2 lineages was rapid; it reached 67% in June, but decreased slightly when the Delta variant emerged in July. The prevalence of B.1.497 decreased from 94.3% to 0.9% in June 2021. 

**Figure 2:**
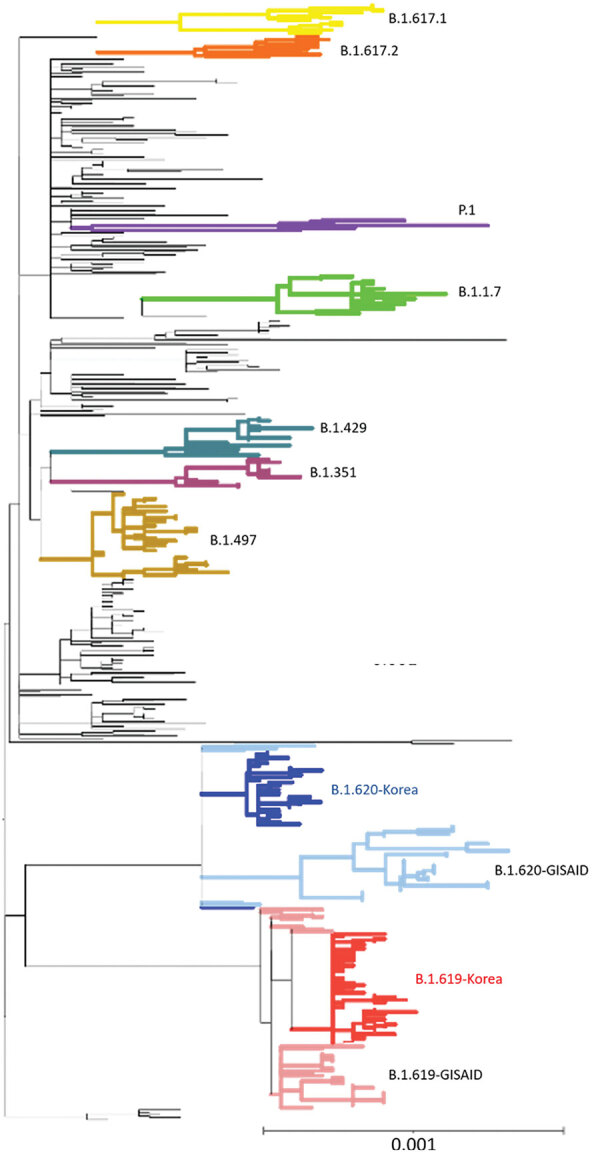
Phylogenetic analysis of severe acute respiratory syndrome coronavirus 2 sequences, South Korea. A total of 457 sequences were used to construct the tree, including 37 sequences of B.1.619 lineage and 36 sequences of B.1.620 lineage from GISAID (https://www.gisaid.org). Each sequence was aligned to the reference sequence (Wuhan-Hu-1, GenBank accession no. NC_045512) using Geneious Prime software (https://www.geneious.com) and then manually trimmed to equal lengths. A maximum-likelihood phylogenetic tree was reconstructed using FastTree version 2.1.9 (http://www.microbesonline.org/fasttree), under the general time reversible plus gamma nucleotide substitution model; the phylogenetic tree was visualized using iTOL (https://itol.embl.de). Four variants of concern (B.1.1.7, B.1.351, P.1, and B.1.617.2), 2 variants of interest (B.1.429 and B.1.617.1), and B.1.497, which were the major lineages of the GH clade in South Korea, are shown. Red indicates South Korea B.1.619 sequences and blue, B.1.620; pink indicates Europe B.1.619 sequences and light blue, B.1.620. Scale bar indicates substitutions per site.

B.1.619 and B.1.620 were identified in imported cases in South Korea in 2021; B.1.619 in a case-patient from Cameroon in February and B.1.620 in cases from Kenya and Malawi in March. The phylogenetic analysis of SARS-CoV-2 sequences isolated in South Korea showed that B.1.619 and B.1.620 were distinct from those in countries in Europe (Figure 2); this finding indicates 1 or very few introduction events for B.1.619 and B.1.620 strains into South Korea, from which strains then spread rapidly. South Korea B.1.619 has been reclassified as B.1.619.1, which has an additional mutation in ORF1ab (K3929R) (*10*).

B.1.620 was prevalent in central Africa and later spread to Europe and the United States through travelers (*10*). We were unable to find previous research on B.1.619 in the literature; however, we assumed that B.1.619 was a prevalent strain in central Africa and later spread to Europe because it has been identified in central Africa, according to information from GISAID.

The B.1.619 and B.1.620 lineages have several characteristic spike protein mutations (Table 1); the E484K mutation, which is present in both Beta and Gamma variants and has been identified as an escape mutation (*11*), is the only shared mutation in both lineages. The mutations in the spike protein, specifically in the RBD, have a strong influence on SARS-CoV-2 pathogenesis; B.1.619 has additional N440K mutations in the RBD and B.1.620 has S447N substitutions. The S477N mutation may evade antibody-mediated immunity (*12*) and increase RBD affinity for ACE2 (*13*). In addition, the N440K mutation might confer resistance to monoclonal antibodies and enhance binding affinity to the ACE2 receptor (*13*,*14*). We observed no other specific mutation in the spike protein in B.1.619. In contrast, B.1.620 carries several mutations and deletions, previously observed individually in VOCs and VOIs (Table 1). The HV69/70Δ, Y144Δ, P681H, and D1118H mutations in the spike protein have been found in the Alpha variant, whereas the LAL242/243/244Δ mutation has been found in the Beta variant. Previously, we found that B.1.619 and 620 have no inhibitory effect on the neutralizing activity in vaccinated or convalescent persons (S.J. Oh et al., unpub. data). However, the combined effect of these mutations on viral pathogenicity and transmissibility needs to be elucidated.

**Table 1:** Amino acid substitutions in severe acute respiratory syndrome coronavirus 2 B.1.619 and B.1.620 lineages, South Korea*

Gene	B.1.497	B.1.619	B.1.620
ORF1a	T265I, P3884L	A2123V, E2607K, del3675/3677, M3752I	T403I, V1991I, del3675/3677
ORF1b	P314L, Q2403L	P314L	P314L, A1215S
S	D614G	I210T, N440K, E484K, D614G, D936N, S939F, T1027I	P26S, del69/70, V126A, del144/145, S477N, E484K, D614G, P681H, T1027I, D1118H
ORF3a	Q57H	None	None
M	None	I82T	None
ORF7a	None	E22D	None
ORF7b	None	None	del14/15
N	None	P13L, S201I, T205I	A220V

*M, membrane glycoprotein; N, nucleocapsid; ORF, open reading frame; S, spike.

## Conclusions

Continuous monitoring of mutations is essential to track potential vaccine efficacy reduction, increased transmissibility, and disease severity. The transmissibility of B.1.619 and B.1.620 and their likelihood to cause more severe infections are not yet confirmed. Preliminary data show that patients who recovered from non-VOC/VOI and vaccinated persons have sufficient neutralizing capacity against these lineages (Table 2). The transmissibility and immune escape of these strains must be investigated further. Continuous genomic surveillance supporting public health response is required to overcome the COVID-19 pandemic.

**Table 2:** Results from neutralization testing of serum from persons who were infected with and vaccinated against severe acute respiratory syndrome coronavirus 2, South Korea*

Serum type	PRNT_50_ GMT (95% CI)		
	G (D614G)	B.1.619	B.1.620
Convalescent, n = 7	79 (35–179)	340 (138–830)	546 (248–1,205)
Vaccinated, n = 7	32 (16–62)	76 (37–155)	584 (303–1,125)

*Test results against G clade (D614G) and B.1.619 and B.1.620 lineages. GMT, geometric mean titer; PRNT_50_, 50% plaque reduction neutralization test.
